# The Importance of Failure: How Doing Impact Surveys That Fail Saves Trachoma Programs Money

**DOI:** 10.4269/ajtmh.20-0686

**Published:** 2020-10-05

**Authors:** Anthony W. Solomon, Pamela J. Hooper, Mathieu Bangert, Upendo J. Mwingira, Ana Bakhtiari, Molly A. Brady, Christopher Fitzpatrick, Iain Jones, George Kabona, Amir B. Kello, Tom Millar, Aryc W. Mosher, Jeremiah M. Ngondi, Andreas Nshala, Kristen Renneker, Lisa A. Rotondo, Rachel Stelmach, Emma M. Harding-Esch, Mwelecele N. Malecela

**Affiliations:** 1 Department of Control of Neglected Tropical Diseases, World Health Organization, Geneva, Switzerland;; 2 Task Force for Global Health, International Trachoma Initiative, Atlanta, Georgia;; 3 Neglected Tropical Disease Control Program, Ministry of Health, Community Development, Gender, Elderly and Children, Dodoma, United Republic of Tanzania;; 4 RTI International, Washington, District of Columbia;; 5 Sightsavers, Haywards Heath, United Kingdom;; 6 Expanded Special Project for Elimination of Neglected Tropical Diseases, World Health Organization Regional Office for Africa, Brazzaville, Congo;; 7 United States Agency for International Development, Washington, District of Columbia;; 8 IMA World Health, Dar es Salaam, United Republic of Tanzania;; 9 Department of International Maternal and Child Health, Faculty of Medicine and Pharmacy, University of Uppsala, Uppsala, Sweden;; 10 Clinical Research Department, London School of Hygiene & Tropical Medicine, London, United Kingdom

## Abstract

Trachoma programs use annual antibiotic mass drug administration (MDA) in evaluation units (EUs) that generally encompass 100,000–250,000 people. After one, three, or five MDA rounds, programs undertake impact surveys. Where impact survey prevalence of trachomatous inflammation—follicular (TF) in 1- to 9-year-olds is ≥ 5%, ≥ 1 additional MDA rounds are recommended before resurvey. Impact survey costs, and the proportion of impact surveys returning TF prevalence ≥ 5% (the failure rate or, less pejoratively, the MDA continuation rate), therefore influence the cost of eliminating trachoma. We modeled, for illustrative EU sizes, the financial cost of undertaking MDA with and without conducting impact surveys. As an example, we retrospectively assessed how conducting impact surveys affected costs in the United Republic of Tanzania for 2017–2018. For EUs containing 100,000 people, the median (interquartile range) cost of continuing MDA without doing impact surveys is USD 28,957 (17,581–36,197) per EU per year, whereas continuing MDA solely where indicated by impact survey results costs USD 17,564 (12,158–21,694). If the mean EU population is 100,000, then continuing MDA without impact surveys becomes advantageous in financial cost terms only when the continuation rate exceeds 71%. For the United Republic of Tanzania in 2017–2018, doing impact surveys saved enough money to provide MDA for > 1,000,000 people. Although trachoma impact surveys have a nontrivial cost, they generally save money, providing EUs have > 50,000 inhabitants, the continuation rate is not excessive, and they generate reliable data. If all EUs pass their impact surveys, then we have waited too long to do them.

## INTRODUCTION

Trachoma causes blindness among the poorest people on the planet.^1^ Trachomatous blindness arises from repeated conjunctival *Chlamydia trachomatis* infection and the eyelid scarring that accumulates from the associated episodes of active (inflammatory) trachoma.^2^ The disease can be eliminated as a public health problem through a four-pronged strategy summarized by the acronym SAFE: surgery (S) for individuals with advanced, blinding disease; antibiotics (A) to clear *C. trachomatis* infection; and facial cleanliness (F) and environmental improvement (E) to reduce ocular *C. trachomatis* transmission.

The A, F, and E components of SAFE are delivered at the evaluation unit (EU) level. Evaluation units are generally populations of 100,000–250,000 people^3^; however, for practical reasons (including expediency at program inception^3^ or the size of local administrative divisions for healthcare management purposes^4^), they are sometimes smaller^4^ or larger^4,5^ than this. Five annual rounds of EU-wide mass drug administration (MDA) of antibiotics active against ocular *C. trachomatis*^6^ are undertaken wherever the prevalence of the active trachoma sign trachomatous inflammation—follicular (TF^7^) in 1- to 9-year-olds is ≥ 30%. Three annual rounds are undertaken wherever the TF prevalence is 10–29.9%. Where the TF prevalence is 5.0–9.9%, the WHO recommends targeted treatment^3^; in recent years, this has been programmatically applied by targeting all EU residents in a single round of MDA^8^; the alternative targeting approaches of offering antibiotics only to individuals,^9^ households,^10–12^ or communities^13^ with active trachoma having been assessed as likely ineffective, impractical at scale, or both. Antibiotic MDA for trachoma should always be accompanied by implementation of F and E; however, the evidence base for F and E is weaker than that for A.^14,15^

Because decisions concerning implementation of SAFE’s A component rest on EU-level prevalence of TF, reliable TF prevalence data are critical. Recently, the global trachoma program has made important investments in appropriately scoping out^16^ and mapping^17–21^ suspected endemic populations using standardized, quality-controlled, and quality-assured approaches.^22–24^ Where baseline TF prevalence is < 5%, the A component is not indicated for trachoma elimination purposes, but the need for interventions to improve access to water and sanitation may be highlighted if current access is suboptimal.^25^ Where baseline TF prevalence is ≥ 5%, A, F, and E are indicated as noted earlier, with impact surveys due 6–12 months after the last planned round of MDA.^26^ Programs use impact survey TF prevalence to determine whether further MDA rounds should be planned. Impact survey TF prevalence < 5% signals an end to MDA for trachoma elimination purposes and the start of a 2-year surveillance period. At the end of those 2 years, a pre-validation surveillance survey is undertaken to ensure that TF prevalence has not recrudesced to ≥ 5%.^26^

Baseline,^21^ impact,^27^ and pre-validation surveillance^28^ surveys are generally all performed using population-based, two-stage cluster sampling.^29^ Survey costs are not insignificant.^30–32^ Trachoma programs are currently undergoing considerable expansion in an effort to achieve the World Health Assembly–endorsed goal of global elimination of trachoma as a public health problem.^33,34^ During a session of the 2018 meeting of the Coalition for Operational Research on neglected tropical diseases (NTDs), discussion focused on the proportion of EUs undergoing trachoma impact surveys in a defined period returning a TF prevalence estimate above the elimination threshold, a proportion sometimes referred to as the failure rate. This rate was said to be higher than the analogous failure rate of lymphatic filariasis transmission assessment surveys, with the implication being that money for trachoma elimination was being inefficiently spent on surveys conducted too soon. It led us to wonder: Is a nonzero failure rate necessarily bad? Here, we demonstrate that the answer is no, by modeling the financial cost of undertaking MDA with and without conducting impact surveys. And, because in this context failure turns out to be helpful, we will henceforth use the more neutral term continuation rate to refer to the proportion of EUs undergoing impact surveys that derive a renewed mandate for MDA.

## MATERIALS AND METHODS

To obtain an impact survey continuation rate of 0% (and thereby save money on monitoring and evaluation by not needing to repeat impact surveys after further rounds of MDA), all impact surveys would need be delayed until a TF prevalence < 5% was thought to be virtually guaranteed. We therefore compared the cost of using impact surveys to guide decision-making on ongoing annual MDA with the cost of simply continuing MDA for another year, considering only the financial costs to the trachoma program in a single year of its operation.

We hypothesized a set of trachoma-endemic EUs in which, at the beginning of the program year, the recommended number of annual rounds of MDA for trachoma elimination purposes had already been completed. Assuming appropriate antibiotics were available, the trachoma program manager could—in theory—choose between two strategies:

(a) undertake an impact survey in each EU, then decide whether to stop or continue MDA in the EU on the basis of the survey outcome; or
(b) undertake another round of MDA in each EU, postponing impact surveys until the next year or beyond.

The cost of strategy (a) would be less than that of strategy (b) when

$$C_{\mathrm{survey}}+\left(P_{\mathrm{continuation}}\times uc_{\mathrm{MDA}}\times \bar{N}\right)<uc_{\mathrm{MDA}}\times \bar{N},$$

where $C_{\mathrm{survey}}$ is the financial cost of an impact survey in one EU, excluding salaries of (but including fieldwork per diems for) survey team members; $P_{\text{continuation}}$ is the impact survey continuation rate; $uc_{\text{MDA}}$ is the unit cost of MDA per person per year excluding the cost of antibiotics and excluding salaries and per diems of distribution team members; and $\bar{N}$ is the mean EU population.

Rearranging, this occurs when

$$P_{\text{continuation}}<\left(\left[uc_{\text{MDA}}\times \bar{N}\right]-C_{\text{survey}}\right)/\left(uc_{\text{MDA}}\times \bar{N}\right)$$

$$\text{or}\;\text{when}\;P_{\text{continuation}}<\left(1-C_{\text{survey}}\right)/\left(uc_{\text{MDA}}\times \bar{N}\right),$$

that is, when the probability of continuation is less than 1 minus the ratio of the impact survey cost to the cost of one round of MDA.

We know that $uc_{\text{MDA}}$ (not just the total cost of MDA) depends on N.^35^ The elasticity of $uc_{\text{MDA}}$ with respect to population size is about −0.5, meaning that for a 10% increase in N, $uc_{\text{MDA}}$ decreases by a mean of 5%. We therefore used a published web-based application (https://healthy.shinyapps.io/benchmark/^35^) to generate estimates of $uc_{\text{MDA}}$ for our analyses. This application requires several parameters to be specified; we assumed an MDA coverage rate of 85% would be achieved in the fourth year of a subnational, annual campaign distributing only antibiotics for trachoma, in a country other than a small-island developing state, using unpaid volunteers to distribute in the community (rather than in schools). Per-capita gross domestic product (GDP) and population density were set at $936 and 123 people per km^2^, respectively: real-world data from the United Republic of Tanzania in 2017. These choices were consistent with the scenario under consideration: WHO recommends that programs achieve at least 80% coverage when undertaking antibiotic MDA^3^; where baseline TF prevalence is 10–29.9%, an impact survey to determine whether or not MDA should continue would normally be undertaken before the fourth round, the United Republic of Tanzania is trachoma endemic,^36^ and so on. The derived $uc_{\text{MDA}}$ estimates were $0.13 (2015 USD; 95% CI: 0.08–0.17), $0.18 (0.11–0.23), $0.20 (0.12–0.26), $0.23 (0.14–0.29), $0.28 (0.17–0.35), and 0.39 (0.24–0.49) for EUs of 500,000, 250,000, 200,000, 150,000, 100,000, and 50,000 people, respectively.

$C_{\mathrm{survey}}$ can also depend on N. The standard error of a prevalence estimate decreases as the sampling fraction increases, allowing smaller sample sizes (for the same level of precision) with smaller underlying populations. In practice, sample sizes do not vary much across the WHO-recommended EU population range: a decrease in EU population size from 250,000 to 100,000 decreases the estimated sample size requirement for 1- to 9-year-olds by only 2.3–3.2%, depending on the other parameters used in the calculation.^29^ What if EUs are constructed to be even smaller than 100,000 people? Although the number of children that should be examined to maintain acceptable precision decreases more steeply, the requirement for those children to be chosen from at least 20 clusters^29^ means that $C_{\mathrm{survey}}$ tends not to fall far. Program data suggest variation in impact survey costs is driven primarily by context-specific expenditure on per diems and transport, with minor economies of scale seen when larger numbers of surveys are undertaken in a single round.^32^ To parameterize $C_{\mathrm{survey}}$ for the current analyses, we used published data compiled from 322 trachoma impact and surveillance surveys conducted in 11 countries; the median per-EU financial cost (in 2017 USD) was $8,298 (interquartile range [IQR]: $6,532–$10,111).^32^ (Surveillance surveys use the same systems and methods and have the same sample size requirements as impact surveys, and the costs of these two survey types do not differ significantly from each other [Mann–Whitney *P* = 0.68; 95% CI for cost difference −$620–$788].^32^ It is therefore appropriate to use the median cost for both survey types combined as the $C_{\mathrm{survey}}$ value in the present analyses.)

Our choices of, for example, per-capita GDP and population density when deriving $uc_{\text{MDA}}$ estimates from https://healthy.shinyapps.io/benchmark/ were necessarily arbitrary and made simply to parameterize the model. We therefore performed a separate analysis using the median $uc_{\text{MDA}}$ determined from 150 observations in 29 studies identified as part of a published systematic review: $0.20 (2015 USD).^35^ We converted $uc_{\text{MDA}}$ estimates from 2015 USD to 2017 USD using an inflation factor of ×1.0342.

We obtained empirical data on $P_{\text{continuation}}$ by compiling global impact survey continuation rates using all surveys completed using Tropical Data (www.tropicaldata.org) in the calendar years 2017 and 2018. We used these inputs, and the same median and IQR $C_{\mathrm{survey}}$ as earlier,^32^ to determine the global per-EU cost of strategies (a) and (b) and the $P_{\text{continuation}}$ at which the costs would equalize, for the same six illustrative $\bar{N}$s.

As a case example, we compared the cost of implementing each strategy within the trachoma elimination program of the United Republic of Tanzania for 2017 and 2018. For this analysis, we used survey and cost data collected retrospectively from the actual surveys implemented during that period.

## RESULTS

From January 1, 2017 to December 31, 2018, Tropical Data supported trachoma programs to complete 538 impact surveys in 25 countries, representing 92% of all impact surveys completed for trachoma globally during that 2-year period.

A total of 170 (32%) of those 538 impact surveys returned estimates of TF prevalence in 1- to 9-year-olds of ≥ 5%, indicating MDA continuation. Continuation rates ranged by country from 0% to 100% of EUs. Fourteen of 25 countries had nonzero continuation rates.

For EUs containing a mean of 100,000 people, the median cost of continuing MDA without doing impact surveys would be USD 28,957 per EU per year, whereas the median cost of doing impact surveys and continuing MDA only where indicated by TF prevalence would be USD 17,564 (Table 1). Continuing MDA without impact surveys becomes advantageous (in financial cost terms) only when the continuation rate exceeds 71%.

**Table 1:** Cost per trachoma-endemic EU in a single programmatic year for (a) undertaking an impact survey in each EU, and then making a decision on whether to stop or continue annual antibiotic MDA for trachoma elimination purposes in that EU on the basis of the outcome; and (b) simply continuing MDA without first conducting impact surveys, and the continuation rate at which financial costs for the two strategies equalize, for different mean EU populations $\left(\bar{N}\right)$

$\bar{N}$	Cost per EU of impact surveys then MDA where indicated*, 2017 USD†	Cost per EU of continuing MDA without first conducting impact surveys‡, 2017 USD†^,^	Impact survey continuation rate at which financial costs equalize, %†
50,000	14,751 (10,503–18,219)	20,166 (12,410–25,337)	58 (47–60)
100,000	17,564 (12,158–21,694)	28,957 (17,581–36,197)	71 (62–72)
150,000	19,715 (13,482–24,507)	35,679 (21,718–44,987)	76 (69–77)
200,000	21,536 (14,474–27,320)	41,368 (24,820–53,778)	79 (73–81)
250,000	23,190 (15,633–29,140)	46,539 (28,440–59,466)	82 (77–82)
500,000	29,809 (19,770–38,241)	67,223 (41,368–87,907)	87 (84–88)

EU = evaluation unit; IQR = interquartile range; MDA = mass drug administration. Calculations were based on the global median (and IQR of) impact survey costs from Stelmach et al.,^32^ the 2017–2018 global Tropical Data impact survey continuation rate of 32%, and the global median (and 95% CIs of) per-person financial cost of MDA from Fitzpatrick et al.,^35^ inflated from 2015 USD to 2017 USD using a factor of ×1.0342.

* Referred to in the text as “strategy (a).”

† First figure in each cell uses the median survey and MDA costs; figures in parentheses reflect the IQR of survey costs and 95% CI of MDA costs.

‡ Referred to in the text as “strategy (b).”

In the United Republic of Tanzania in the 2 years from January 1, 2017 to December 31, 2018, 20 impact surveys were required. The continuation rate was 6/20 (30%). The cost of not doing impact surveys and just undertaking MDA in each of those 20 EUs (strategy [b]) would have been $409,721. The cost of the process actually performed—undertaking impact surveys and implementing MDA only where indicated (strategy [a])—was $307,790, a saving of $101,931 (25%, Table 2). Based on the local cost of MDA in 2017–2018 ($0.0981 per person, Table 2), this is equivalent to the cost of undertaking MDA for more than one million people.

**Table 2:** Retrospective estimate of costs to the United Republic of Tanzania’s trachoma elimination program of either (a) undertaking an impact survey in each EU in which one was due in 2017 or 2018 and then deciding whether to stop or continue annual antibiotic MDA for trachoma elimination purposes in that EU on the basis of the outcome; or (b) simply continuing MDA without first conducting impact surveys

District	EU	Year of impact survey	Cost of impact survey (USD)	Estimated population at the time of impact survey	Cost of one round MDA (USD), if needed	Strategy (a)		Strategy (b)	Annual saving achieved using strategy (a) rather than (b)
						Trachomatous inflammation—follicular prevalence category at impact survey (MDA needed?)	Total cost of impact survey + MDA in year of impact survey	Cost of MDA (no impact survey)	
Nkasi	Nkasi	2018	8,719.32	318,958	28,884.11	< 5% (no)	8,719.32	28,884.11	20,164.79
Kalambo	Kalambo	2018	7,610.23	235,589	21,351.88	5–9.9% (yes)	28,962.11	21,351.88	−7,610.23
Ngara	Ngara	2018	9,501.14	386,638	16,728.38	< 5% (no)	9,501.14	16,728.38	7,227.24
Songwe	Songwe	2018	6,546.59	153,820	10,947.88	5–9.9% (yes)	17,494.47	10,947.88	−6,546.59
Chunya	Chunya	2018	8,673.86	163,315	12,595.67	< 5% (no)	8,673.86	12,595.67	3,921.80
Bahi	Bahi	2018	6,382.95	251,080	22,565.15	< 5% (no)	6,382.95	22,565.15	16,182.20
Chemba	Chemba	2018	8,401.14	267,014	32,301.05	5–9.9% (yes)	40,702.19	32,301.05	−8,401.14
Liwale	Liwale	2018	7,610.23	96,427	18,195.65	< 5% (no)	7,610.23	18,195.65	10,585.42
Longido	Longido	2018	10,616.59	144,410	18,468.33	5–9.9% (yes)	29,084.92	18,468.33	−10,616.59
Monduli	Monduli	2018	10,616.59	186,477	20,565.78	< 5% (no)	10,616.59	20,565.78	9,949.20
Ngorongoro	Ngorongoro	2018	11,611.66	204,487	24,463.96	10–29.9% (yes)	36,075.61	24,463.96	−11,611.66
Kalambo	Kalambo	2017	10,341.64	235,589	26,436.67	5–9.9% (yes)	36,778.31	26,436.67	−10,341.64
Kilindi	Kilindi	2017	8,788.41	258,372	26,248.10	< 5% (no)	8,788.41	26,248.10	17,459.69
Itigi	Itigi	2017	8,427.59	127,680	14,067.43	< 5% (no)	8,427.59	14,067.43	5,639.83
Manyoni	Manyoni	2017	8,597.45	213,010	20,933.81	< 5% (no)	8,597.45	20,933.81	12,336.36
Kongwa*	Kongwa south	2017	6,973.86	197,409	23,779.53	< 5% (no)	6,973.86	23,779.53	7,970.53
Kongwa*	Kongwa north	2017	8,835.14	113,042		< 5% (no)	8,835.14		
Chamwino*	Chamwino south	2017	8,256.36	155,647	39,564.67	< 5% (no)	8,256.36	39,564.67	22,820.48
Chamwino*	Chamwino north	2017	8,487.73	147,574		< 5% (no)	8,487.73		
Meatu	Meatu	2017	8,821.82	321,781	31,622.54	< 5% (no)	8,821.82	31,622.54	22,800.72
Totals			173,820.28	4,179,319	409,720.59	–	307,709.06	409,720.59	101,930.53

EU = evaluation unit; MDA = mass drug administration.

* Districts divided into two EUs for impact survey purposes.

## DISCUSSION

A trachoma elimination program in which all impact surveys estimate TF to be < 5% has waited too long to do them and will in the meantime have incurred unnecessary intervention costs. Doing surveys to determine whether MDA is still needed is generally cheaper than just continuing MDA. The qualification generally is needed in that statement because the certainty of the conclusion would diminish when survey costs or survey continuation rates rise, when MDA costs fall, or for mean EU populations less than 50,000. This observation underlines the merit of the 2010 WHO recommendation that trachoma program EUs be framed as populations of 100,000–250,000 people,^3^ a recommendation that tried to balance considerations relating to disease control, ethics, existing administrative divisions, local politics, and program costs. The cost element in that balance was intuitive; the present analyses allow the intuition to be formally tested—and found to be correct. Although it may not be straightforward for health ministries to combine local administrative areas with small populations into a single EU for trachoma elimination purposes, where local administrative areas have very small populations, doing so probably increases the cost efficiency of the elimination program.

Understanding that impact surveys save money even when some EUs fail them is important. The corollary—that a nonzero continuation rate should be welcomed—is even more important because it can seem counterintuitive. Spending money on monitoring and evaluation can make program managers and partners uncomfortable. The proportion of funds that can be spent on monitoring and evaluation is capped in some program grants, and the view is sometimes expressed that funds allocated to these activities would be more productively used for disease control. This ignores the fact that programs must periodically reconfirm the presence of the disease being controlled. We have shown in this article that, assuming EUs are larger than 50,000, and the continuation rate and unit costs are not too dissimilar from those included in our models, surveys save money for the program even in the year that they are performed. The reason for the saving is that MDA is delivered only to people who actually need it.

We note that survey data quality is paramount.^23^ Misclassifying EUs by conducting surveys with inadequate sample sizes, inappropriate sampling strategies, unstandardized graders, or inappropriate analysis methods invalidates any consideration of the use of surveys to improve cost efficiency. We note also that specific local conditions may fall outside the range of our illustrative calculations. The formulas and code (provided here: https://github.com/mathi-eu/tis-failure) will allow replication or contextual adjustment.

Our analyses are relatively simple. They have several inherent limitations: the first of which is that they include only the financial costs to the program. We ignored the economic costs of the time of survey field-workers, time of survey participants, time of antibiotic distribution staff, and time of antibiotic recipients. Second, the $uc_{\text{MDA}}$ estimates that we used may be imperfect. They were derived from studies across multiple NTDs^35^ because this allowed us to adjust for the elasticity of $uc_{\text{MDA}}$ with changing EU population size. Our $uc_{\text{MDA}}$ estimates also excluded per diems of MDA teams; by contrast, we included the cost of per diems of survey fieldwork teams in our $C_{\mathrm{survey}}$. Actual published $uc_{\text{MDA}}$ estimates for trachoma elimination programs range from $0.25 (Mali; year 2000–2002) to $1.37 (South Sudan; 2010).^37–39^ Undertaking a preparatory population census or return household visits to maximize population coverage makes $uc_{\text{MDA}}$ more expensive.^37^ Third, for the sake of simplicity and robustness, we ignored the finite population correction factor that can be used to reduce survey sample sizes for EUs with smaller populations^29^; this would have reduced our $C_{\mathrm{survey}}$. Fourth, again for the sake of simplicity, we limited our analyses to costs in the program year in which impact surveys are due. This disregarded the cost of ongoing annual MDA in subsequent years when the likelihood of it being necessary for trachoma elimination purposes would continue to wane. Fifth and perhaps most critically, we ignored externalities that would need to be accounted for were annual antibiotic MDA to continue indefinitely without regular affirmation of need. These externalities include a perceived lack of progress toward public health goals, with resulting loss of stakeholder confidence^40^; economic and environmental costs of manufacture and shipment of antibiotics; and possible emergence of antimicrobial resistance.^41,42^ Each of these five limitations is inherently conservative: had we incorporated the relevant considerations in our model, the case for doing more impact surveys rather than fewer would have been even more compelling. This article, then, presents the short-term financial rationale for undertaking high-quality impact surveys at the time when they are due.

The potential externalities of completely interrupting transmission of ocular *C. trachomatis*,^43–45^ reducing transmission of genital *C. trachomatis*,^46^ improving child survival,^47,48^ or eradicating yaws^49–51^ might, of course, strengthen the argument for continuing MDA. (Evidence for each of these possible outcomes of MDA is incomplete.) Similarly, if early discontinuation of MDA was strongly associated with later recrudescence of active trachoma, then both the benefit of continuing MDA for longer and the eventual financial cost of not doing so would be likely to increase. We also disregard the externalities involved in undertaking integrated surveys^52^ and integrated disease control and elimination programs,^53^ which might significantly affect the economic equation.

An additional limitation of this study comes in considering how to apply its conclusions. Programs that have conducted impact surveys for only a few EUs will have continuation rates that are liable to change profoundly as more local experience accrues. And perhaps more importantly, use of a high continuation rate as the lone metric to justify omitting impact surveys would be to ignore the important nonfinancial considerations outlined earlier.

It is worth noting that the counterfactual scenario that we presented—of simply continuing MDA for trachoma until impact survey failure becomes extremely unlikely—would be difficult to put into practice, for two reasons. First, to make donated drug available, the International Trachoma Initiative (which serves as the steward for Pfizer’s [New York, NY] Zithromax^®^ donation) requires program managers to provide evidence of ongoing need for MDA in the form of high-quality TF prevalence data.^8^ Without the donation, continuation of MDA would require azithromycin to be purchased, increasing $uc_{\text{MDA}}$. Second, we do not yet know enough to be able to confidently predict the number of annual rounds of MDA required to reduce the TF prevalence in any particular EU to < 5%. Many variables, including parameters related to water, sanitation, eye-seeking flies, facial cleanliness, and population density,^2^ are associated with TF prevalence and could conceivably modify the effect of antibiotic treatment; the F and E components of SAFE attempt to influence some of these parameters in parallel with antibiotic treatment,^54^ but have unknown effectiveness. The influence of EU-level antibiotic coverage (let alone heterogeneity of coverage spatially or by age and gender) is unclear.^55,56^ Given increasing concern about global antimicrobial resistance and the need to preserve the utility of macrolides,^57^ taking steps to ensure that antibiotic MDA occurs only where and when justified is critical, particularly if it also allows scarce global health dollars to be directed to other areas of need.

Although our analysis was driven by trachoma program data, similar considerations apply to other disease elimination efforts in which mass interventions are undertaken. Other NTD programs provide an immediate parallel.^58^ As a touchstone for the 2030 Agenda for Sustainable Development, the movement to control, eliminate, and eradicate NTDs has few equals, such is the impact of the diseases on the impoverished populations in which they thrive.^59^ Good data are critical for all.^60^ We hope that this article will encourage ongoing support for both interventions against these diseases and the high-quality monitoring and evaluation needed to guide implementation.
